# Curing Kinetics and Thermal Stability of Epoxy Composites Containing Newly Obtained Nano-Scale Aluminum Hypophosphite (AlPO_2_)

**DOI:** 10.3390/polym12030644

**Published:** 2020-03-12

**Authors:** Farimah Tikhani, Shahab Moghari, Maryam Jouyandeh, Fouad Laoutid, Henri Vahabi, Mohammad Reza Saeb, Philippe Dubois

**Affiliations:** 1 School of Chemical Engineering, College of Engineering, University of Tehran, Tehran 11155-4563, Iran; farimah.tikhani@gmail.com (F.T.); shahabmoghari42@gmail.com (S.M.); 2 Université de Lorraine, CentraleSupélec, LMOPS, F-57000 Metz, France; maryam.jouyande@gmail.com; 3 Laboratory of Polymeric & Composite Materials, Materia Nova Research Center, Place du Parc 23, B-7000 Mons, Belgium; fouad.laoutid@materianova.be; 4 Department of Resin and Additives, Institute for Color Science and Technology, Tehran 16765-654, Iran; 5 Laboratory of Polymeric and Composite Materials (LPCM), Center of Innovation and Research in Materials & Polymers (CIRMAP), Health and Materials Research Institutes, University of Mons, Place du Parc, 23, B-7000 Mons, Belgium

**Keywords:** *cure index*, epoxy nanocomposite, nano-scale aluminum hypophosphite, cure kinetics, thermosetting resins

## Abstract

For the first time, nano-scale aluminum hypophosphite (AlPO_2_) was simply obtained in a two-step milling process and applied in preparation of epoxy nanocomposites varying concentration (0.1, 0.3, and 0.5 wt.% based on resin weight). Studying the cure kinetics and thermal stability of these nanocomposites would pave the way toward the design of high-performance nanocomposites for special applications. Scanning electron microscopy (SEM) and transmittance electron microscopy (TEM) revealed AlPO_2_ particles having domains less than 60 nm with high potential for agglomeration. *Excellent* (at heating rate of 5 °C/min) and *Good* (at heating rates of 10, 15 and 20 °C/min) cure states were detected for nanocomposites under nonisothermal differential scanning calorimetry (DSC). While the dimensionless curing temperature interval (ΔT*) was almost equal for epoxy/AlPO_2_ nanocomposites, dimensionless heat release (ΔH*) changed by densification of polymeric network. Quantitative cure analysis based on isoconversional *Friedman* and *Kissinger* methods gave rise to the kinetic parameters such as activation energy and the order of reaction as well as frequency factor. Variation of glass transition temperature (*T_g_*) was monitored to explain the molecular interaction in the system, where *T_g_* increased from 73.2 °C for neat epoxy to just 79.5 °C for the system containing 0.1 wt.% AlPO_2_. Moreover, thermogravimetric analysis (TGA) showed that nanocomposites were thermally stable.

## 1. Introduction

Epoxy resin, as a versatile high-performance and thermosetting material with acceptable chemical and corrosion resistance and low shrinkage, has been shown to be applicable in industrial fields like adhesives and coatings [1,2]. However, there are microstructural requirements to be met in order to make epoxy resins resistant in harsh environments. Exploring the structure–property relationship in thermoset polymer nanocomposites makes possible the development of high-performance materials [3]. There are several foundational works on the optimization of the microstructure of thermoset nanocomposites with the aim of achieving better properties [4,5,6]. Typically, cross-linking density is recognized as the key parameter determining the efficiency of microstructure modification [7]. The analysis of cure is a fundamental concept for tracing the progress of the system toward nanostructure formation within thermoset materials [8,9]. Recently, *Cure Index* was proposed as a comprehensive dimensionless criterion for taking a quick qualitative image of cure, simply projecting contributions of the type, surface chemistry, size, and morphology of additives to the crosslinking reaction taking place in thermoset composites [10]. Nevertheless, it cannot be denied that the uniform dispersion of micro/nanoparticles is the prerequisite for developing a thermoset composite with excellent performance [11,12,13].

Application of inorganic phosphorus-based materials has been a trend of modification, especially in polymers containing oxygen, in order to improve their thermal stability [14,15]. The addition of phosphoric acid leads to the release of water at elevated temperatures, as well as the catalyzation of dehydration reactions, forming carbocations as a crosslinking aid [16]. Although the synergistic thermal stability effects of phosphorus compounds in epoxy thermoset resins have been reported previously [17], the current understanding of cure behavior and kinetics of phosphorus-containing epoxy composites and nanocomposite coatings lags behind their rapid development in industrial applications. A comprehensive study on the contributions of nano-scale types of phosphorus materials in low concentrations is required, since the agglomeration of nanoparticles at higher loadings may prevent the cure conversion from progressing [18,19].

Aluminum hypophosphite (AHP), AlPO_2_, hereafter simply referred as AHP, is a highly reactive phosphorous-based compound because it contains phosphorous at low oxidation degree. Thanks to its high reactivity, AHP has been used as a flame retardant additive for polymers [20,21] at low incorporation content. However, AHP is thermally stable up to 300 °C, and its thermal degradation leads to the release of toxic phosphine. For this reason, its use in some technical polymers is limited in order to avoid phosphine emission during melt processing. 

Epoxy resin is well adapted for hosting AHP as a flame retardant agent. In fact, epoxy processing temperature does not exceed 300 °C, and a relatively low rate of incorporation is required, since AHP is highly reactive. However, the effect of the incorporation of AHP into epoxy and the mechanisms of its performance in the network formation have been the subject of only a limited number of works. For example, Shi et al. [22] suggested that the introduction of 2 wt.% of AHP into unsaturated polyester resin in combination with a multivalent phosphorus precursor might improve crosslinking reactions. However, to the best of our knowledge, no report has ever been published on the role of AHP nano-scale particles in the curing behavior and crosslinking degree of epoxy resins. 

In this work, new AHP nanoparticles were prepared, and their role in epoxy curing behavior and structure–property relationships of their nanocomposites was discussed. First, AHP microparticles were processed to obtain nano-scale particles, and then they were characterized by scanning electron microscopy (SEM) and transmittance electron microscopy (TEM). Low concentration epoxy nanocomposites of AHP (0.1, 0.3 and 0.5 wt.% based on epoxy weight) were prepared from a solution of AHP in ethanol to avoid solubilization. Differential scanning calorimetry (DSC) at various heating rates of 5, 10, 15 and 20 °C/min was performed to evaluate cure behavior and kinetics in terms of enthalpy of cure, curing temperature window, featured in *Cure Index* and based on a general protocol [10,23]. The samples were designated with cure labels including *Poor*, *Good* and *Excellent*. Additionally, quantitative cure analysis was carried out based on kinetic parameters such as activation energy and order of reaction, as well as the frequency factor based on the *Friedman* and *Kissinger* isoconversional methods. Glass transition temperature (*T_g_*) was used as an indicator of molecular mobility and interactions of constituents in the system, and was measured by reciprocal scanning of fully cured samples at heating rate of 10 °C/min. Finally, the thermal properties of the nanocomposites were evaluated by thermogravimetric analysis (TGA). 

## 2. Materials and Methods

### 2.1. Materials

AHP microparticles were purchased from Hutong Global Co., Ltd. (Tianjin, China). Bisphenol A diglycidyl ether (EPON™ Resin 828) epoxy resin with epoxide equivalent weight (EEW) of 450–550 g/Eq. was manufactured by Hexion (Columbus, OH, USA). The curing agent was Epikure™ F205 based on isophorone diamine provided by Hexion (Columbus, OH, USA), with a hydrogen equivalent weight (HEW) value of 105 g/eq and an ambient viscosity of 500–700 mPa.s. 

### 2.2. AHP Nanoparticles Preparation

A two-step milling process was conducted on the as-received AHP particles by SDTech company, SDTech Nano France (Alès, France). To facilitate nanometer-scale fulfilment, an intervening size reduction step was required to achieve the particle size of 1 µm. For this aim, grinding balls with diameter of 1 mm and a grid mesh with the size of 400 µm were used (microbeads Netzsch Labstar mill). During the second step, the nanoparticles were obtained by using smaller grinding balls and grid mesh (diameter of 200 µm and 100 µm, respectively). The second milling step was performed in ethanol in order to avoid solubilization of AHP particles. In the whole downsizing process, the filling ratio of grinding balls and rotor speed were kept at 90% and 3000 rpm, respectively.

### 2.3. Preparation of Epoxy/AHP Nanocomposites

Nanocomposites of epoxy containing AHP nanoparticles in different concentrations of 0.1, 0.3, and 0.5 wt.% were prepared in order to characterize network formation. In order to do so, proper amounts of AHP/ethanol (9%) solution and epoxy resin were mixed and sonicated for 5 min. Then, the mixture was subjected to mechanical mixing for 20 min at speed of 1500 rpm. To evaporate the excess solvent, mixture was placed in an oven at 45 °C for 24 h. Finally, the hardener was added to the resulting mixtures at 2:1 resin:hardener stoichiometric ratio at ambient temperature and was thoroughly stirred for 3 min to make homogeneous nanocomposite samples.

### 2.4. Characterization Methods

#### 2.4.1. Electron Microscopy (SEM and TEM)

Microscopic observations of AHP microparticles were performed by scanning electron microscopy (SEM) analysis using a JEOL JSM 6100 apparatus (JEOL Ltd., Tokyo, Japan) at 5 keV. Samples were coated with gold prior to SEM observations. AHP nanoparticle size was evaluated by Transmission electron microscopy (TEM, Eindhoven, The Netherlands) employing a Philips CM100 microscope with an acceleration voltage of 30 keV.

#### 2.4.2. Nonisothermal Differential Scanning Calorimetry (DSC)

Cure reactions of the blank epoxy and nanocomposite samples were studied by nonisothermal DSC conducted using DSC 4000 PerkinElmer device (Waltham, MA, USA). Samples of 12 mg was weighted and placed in an aluminum pan which were held in a refrigerator after preparation, were contingent upon DSC, test in a nitrogen environment with flow rate of 20 mL/min at four different heating rates of 5, 10, 15, and 20 °C/min. The temperature range was chosen to be between 15 and 250 °C in order to cover up throughout the curing process. The reciprocal heating, cooling, and reheating at 10 °C/min were used for determination of *T_g_*. 

As mentioned previously, additive presence in thermoset resin can hinder cure reaction or facilitate it, depending on the characteristics of nanomaterial. This effect can be evaluated by calculation of fractional extent of conversion as a function of reaction time, which is defined as below:

(1) $$\alpha =\frac{\Delta H_{T}}{\Delta H_{\infty }}$$

where Δ*H_∞_* is the total heat of cure reaction and Δ*H_T_* is the enthalpy of the reaction up to a specific temperature *T*. 

#### 2.4.3. Thermogravimetric Analysis (TGA)

In order to study the thermal stability of fully cured neat epoxy and its nanocomposites containing 0.1, 0.3 and 0.5 wt.% AHP, TGA was conducted on a STA-6000 instrument (Perkin-Elmer, Norwalk, CT, USA) within a temperature range of 25–600 °C with a heating rate of 10 °C/min in N_2_ atmosphere with a flow rate of 20 mL/min. For this purpose, about 8 mg of samples were weighted and placed in a ceramic crucible. This analysis was performed in order to assess thermal stability of epoxy/AHP nanocomposites. The temperature of 5% and 10% weight loss, *T*_5%_ and *T*_10%_, respectively, and the char residues, were extracted from TGA data.

## 3. Results and Discussion

### 3.1. Characterization of AHP Nanoparticles

**Figure 1:**
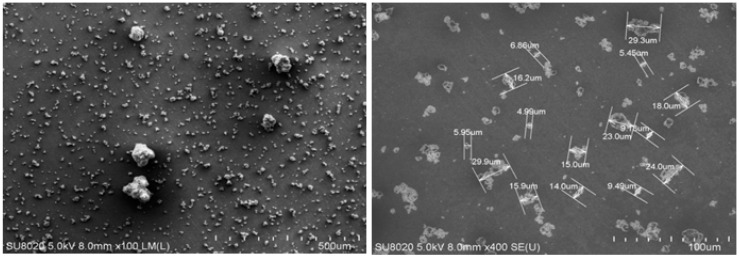
SEM micrographs of aluminum hypophosphite (AHP) microparticles before milling.

Figure 1 shows SEM micrographs of AHP particles before the milling process. In the images, the aggregation of pristine AHP particles can be observed to fall within a size range of 5–30 μm through SEM. 

**Figure 2:**
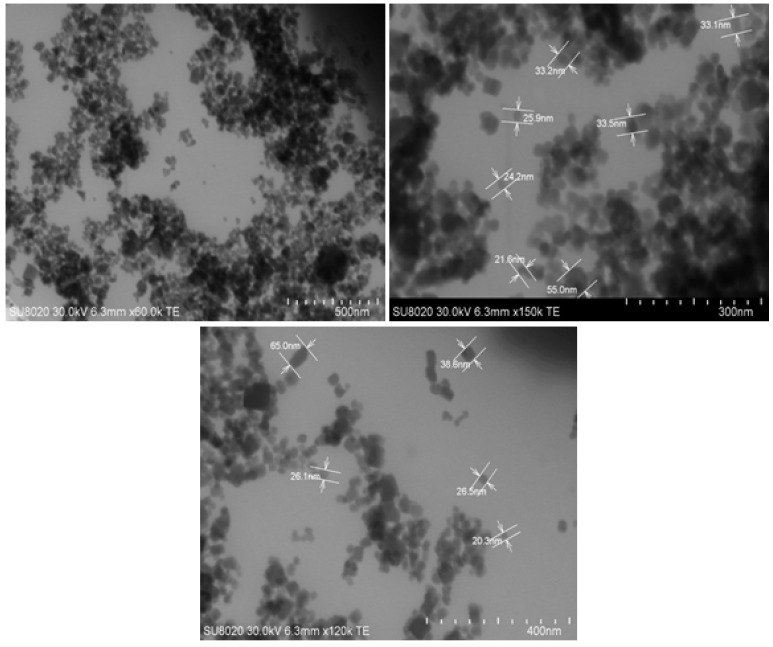
TEM micrographs of AHP nanoparticles obtained by milling.

It was expected that by performing the two-step milling process on the material, particles soud appear in nano-scale through microscope observation. The successful downsizing of AHP allowed obtaining nanoparticles with a diameter lower than 60 nm, confirmed by TEM monitoring (Figure 2). Higher surface area due to size reduction caused more agglomeration due to high surface energy. This is why the images mostly indicate the populations of nanoparticles.

### 3.2. Qualitative Cure Analysis

**Figure 3:**
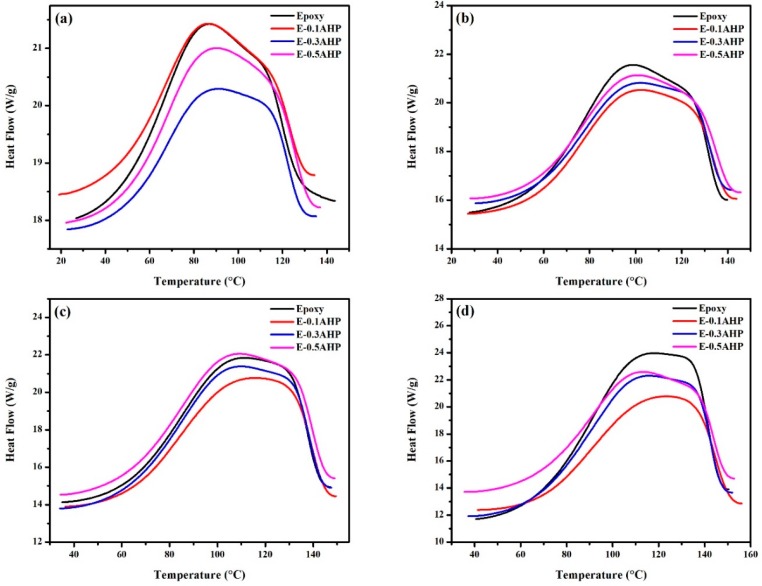
Nonisothermal DSC thermograms of epoxy and epoxy/AHP nanocomposites at heating rates of: (**a**) 5, (**b**) 10, (**c**) 15 and (**d**) 20 °C·min^−1^.

For the evaluation of cure potential of epoxy/amine system intermediated by AHP nanoparticles in different concentrations, nonisothermal DSC was employed at heating rates of 5, 10, 15 and 20 °C/min (Figure 3). The broad peak appeared irrespective of sample type in the thermograms of samples, which can be considered as a double-peaked curve, where the smaller shoulder overlaps with the main temperature peak. Meanwhile, the typical autocatalytic epoxy ring opening reaction commonly leads to a unimodal exothermic peak [18,24]. Therefore, secondary reactions take place in parallel, making the cure kinetics complex [25]. Since this behavior is observed for neat epoxy as well, the shoulder cannot be attributed to the contribution of nanoparticles in the cure reaction; rather, it belongs to the epoxy system. The chemical structure of epoxy used in this study contains hydroxyl groups on its own backbone, which is one of the reactive functionalities capable of opening the epoxide group. Therefore, in the early stages, the curing progress is governed by the chemical reactions of the oxirane with primary and secondary amines of the curing agent. The shoulder is more salient at higher temperatures, when epoxy participates in etherification reactions with hydroxyl groups present in the system that originated both from the previous stage and from the epoxy backbone structure. This reaction is inferior, or of lower likelihood, at low temperatures [26,27]. Increasing the heating rate to higher values shifted cure characteristics, including onset (*T_onset_*), peak (*T_p_*) and final (*T_endset_*) temperatures, to higher values (Table 1) as a result of the fact that the shortage of time for cure moieties to react at high heating rates is compensated for at higher temperatures [28]. 

To make an inclusive assessment, we need a powerful measure that integrates all the factors affecting the whole cure process into a simple criterion, known as *Cure Index* (*CI*). The applicability and versatility of the *CI* have been repeatedly proved in previous works [29,30]. To calculate such a measure, one should consider the following equations [10]:

(2) $$\Delta H^{*}=\frac{\Delta H_{C}}{\Delta H_{Ref}},$$

(3) $$\Delta T^{*}=\frac{\Delta T_{C}}{\Delta T_{Ref}},$$

(4) $$CI=\Delta H^{*}\times \Delta T^{*},$$

where ΔH* and ΔT* are dimensionless enthalpy and temperature interval for the complete curing process, as determined from the values calculated for epoxy nanocomposites (Δ*H_C_* and Δ*T_C_*) divided by their corresponding values for the neat epoxy (Δ*H_Ref_* and Δ*T_Ref_*), respectively. The defined parameters are calculated at all heating rates and are reported in Table 1.

Placing the data in Table 1 under scrutiny, it has been demonstrated that the introduction of AHP nanoparticles to the epoxy matrix has contributed to cure reactions of epoxy resin, for all the *CI* values are laid in *Good* and *Excellent* cure states. However, the temperature window of the cure is almost unvaried for samples cross-linking at the same heating rate, the incorporation of AHP nanocomposites could intensify the release of heat throughout the duration of the reaction, in comparison to the reference sample. Liu et al. [31] proposed some evidence, such as thermal stability improvement, that confirmed the interactions between a metal phosphinate additive and epoxy matrix and their compatibility. The aluminum cation present in phosphorus precursors can also participate in the epoxy ring-opening reaction as a consequence of complexing with oxygen as a Lewis acid [32]. It should be mentioned that the presence of AHP nanoparticles did not change the kinetics of cure, and only provided more active sites thanks to aluminum cations that could result in a denser network. Low concentrations of nanoparticles facilitate the dispersion of nanoparticles in the matrix and prevents physical limitations such as viscosity increase or dilution effect from occurring. At very low concentrations (0.1 wt.%), the minimized amount of agglomeration, and at higher concentrations (0.3 and 0.5 wt.%), numerous active sites introduced to the system are the reasons why *Excellent* and *Good* cure states are achieved. It should be added that at low heating rate all nanocomposites unconditionally took the *Excellent* label, and possessed the characteristic of completely curing the thermosetting networks [33,34]. 

**Figure 4:**
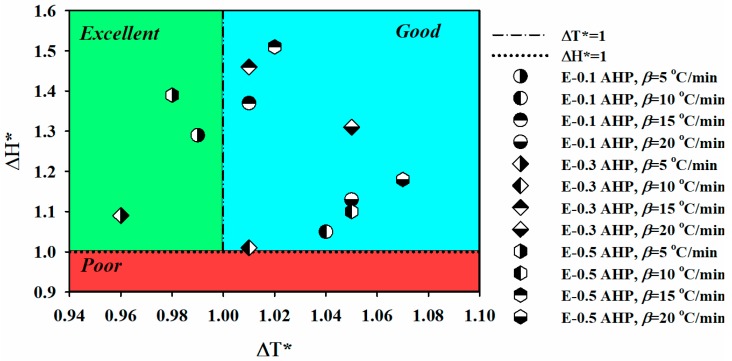
Graphical representation of cure state of epoxy nanocomposites, ΔH* and ΔT* are dimensionless enthalpy and temperature interval for the complete curing process.

Figure 4 graphically exhibited the aforementioned cure states of epoxy nanocomposites for deeper assessment. The cure states of epoxy nanocomposites containing 0.1, 0.3 and 0.5 wt.% of AHP nanoparticles are presented in Figure 4 based on the *CI* in the plot of the dimentionless ΔH* versus dimentionless ΔT*. The placement of the *CI* in the green region, which is representative of an *Excellent* cure (ΔT* *< CI* < ΔH*), demonstrates the importance of providing enough time and energy for molecular collision in the system for all the samples cured at heating rate of 5 °C/min. As the heating rate increases and consequently the equilibrium time decreases, the cure moieties encounter serious difficulties in reaching each other for reaction, resulting in a *Good* cure state (*CI* > ΔH*) instead of an *Excellent*. It can be realized that the contribution of nanoparticles to the cure performance of the system is very bold; such that, even at high heating rates, the cure quality is improved regardless of the concentration of nanoparticles in the matrix, and no system with a *Poor* cure state (*CI* < ΔT*) was observed to be in the red zone [35,36,37].

### 3.3. Quantitative Cure Analysis

It is recommended that a two-step quantitative cure analysis be performed on thermoset nanocomposites complementarily with a qualitative cure analysis in order to achieve a deeper and more detailed understanding of additive/polymer interactions and cure progress [38]. Cure behavior and kinetics are two different but highly relevant parts of quantitative cure analysis that can be investigated in order to predict the ultimate properties of the thermoset nanocomposite. Cure behavior reveals the variations in the reaction rate to be dependent on the amount, particle size and reactivity of the additives, while in cure kinetics, model-fitting or isoconversional methods tend to be applied to determine the kinetic parameters.

**Figure 5:**
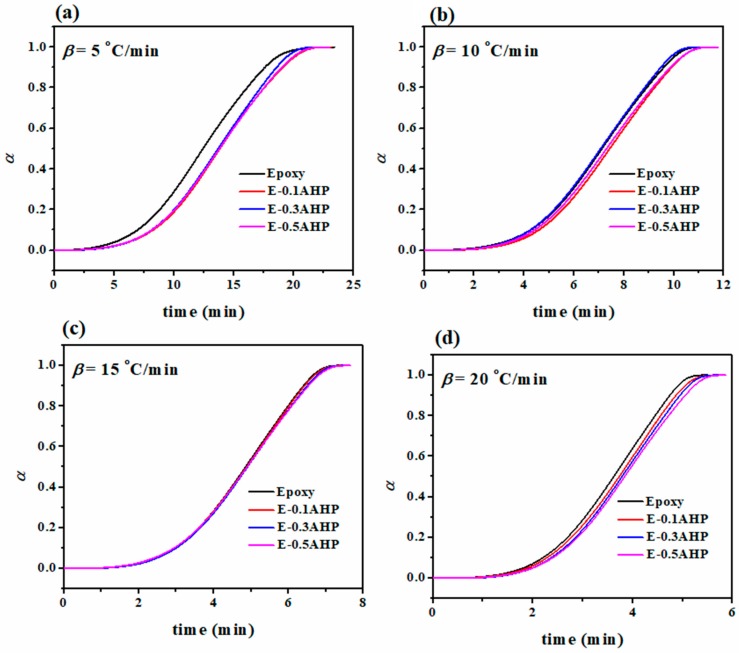
Fractional extent of conversion vs. time for epoxy and AHP/epoxy nanocomposites at heating rates of (**a**) 5, (**b**) 10, (**c**) 15 and (**d**) 20 °C/min.

The *α*-time curves in Figure 5 indicate that cure conversion progress in all samples follows a similar trend to that of the reference sample. The results give an image of the dominant mechanism of reaction which appears to be autocatalytic due to the sigmoidal shape of the curve [39]. The retardation in the preliminary phase of the cure is explained by increased viscosity as a result of nano-additives, which hinders the accessibility of cure species [40]. From a molecular point of view, the numerous active sites participating in network formation thanks to AHP nanoparticles need a longer time interval for the ring opening reaction, since they have a more prominent role in the diffusion-control stage of the process. This effect is boosted at low heating rates of 5 °C/min due to the larger amount of time required. On the other hand, by increasing the heating rate, this effect is diminished as a result of the higher kinetic energy per molecule, which increases the mobility and, consequently, the number of collisions among curing moieties. 

Based on these evaluations, one of the best ways to determine kinetic parameters such as activation energy (*E_a_*) and order of reaction is to apply independent isoconversional methods that do not require model-fitting for calculation of *E_a_* and Frequency factor (*A*) [23]. At a given *α*, these methods assume that the reaction rate is determined purely in terms of temperature (*T*). Any new factor kinetically stimulating the system, such as the incorporation of filler into the matrix, would emerge as an alteration of the behavior of activation energy relative to the extent of the reaction. Among all of the proposed isoconversional methods, this behavior can be determined by approximation-free *Friedman* differential model in combination with the *Kissinger* integral method (explained in Appendix A.1 and Appendix A.2). 

**Figure 6:**
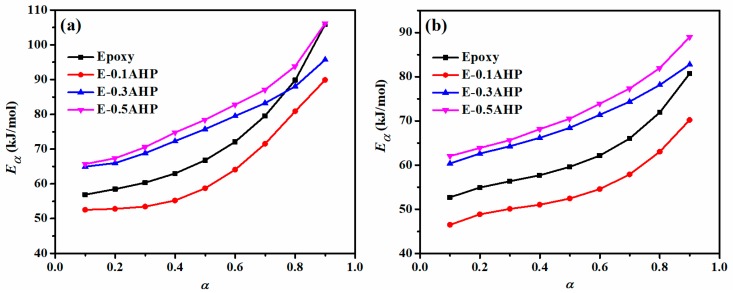
Evolution of activation energy as a function of the extent of cure reaction for epoxy and AHP/epoxy nanocomposites estimated by (**a**) differential *Friedman* and (**b**) integral *Kissinger* methods.

Figure 6 demonstrates correlation of *E_a_* and *α* as calculated by the *Friedman* and *Kissinger* approaches for epoxy and AHP/epoxy nanocomposites. The increasing evolution of activation energy with respect to cure conversion in the range of 0.1 to 0.9 is indicative of the transition from a rapid chemical-control to a gentle diffusion-control phase of the reaction, leading to an increase in the activation energy required for epoxy ring opening at high viscosity, and the difficult accessibility of reaction constituents following the vitrification point, which is the point at which the glass transition temperature of system exceeds the curing temperature [41,42].

In light of the contribution made by nanoparticle content in the nanocomposite, it can be seen from Figure 6 that the presence of very low amounts of AHP nanoparticles in epoxy resin (0.1 wt.% of polymer) caused a significant decrease in *E_a_* throughout the whole range of cure conversion in comparison to unfilled epoxy, which exhibits a facilitated cure reaction and molecular mobility. On the contrary, a higher concentration of nanoparticles requires a greater amount of energy in order to initiate the reaction due to aggregate formation and the higher viscosity of the system. Although the behavior described can be predicted by both isoconversional methods, the values of *E_a_* calculated by the *Friedman* model are slightly higher than those calculated using the *Kissinger* model.

To answer the question as to “whether or not the introduction of nanoparticles has affected the auto-catalytic/non-catalytic ratio of curing mechanism”, we need to determine a reaction model by applying valid approaches like the *Malek* method when the activation energy is determined. The detailed mathematical relations are described in the Appendix B.1 and Appendix B.2 sections. This method takes advantage of the maximum points of *y*(*α*) curve (*α_m_*) and *z*(*α*) curve (*α_p_*^∞^), together with the peak conversion in DSC thermograms (*α_p_*) as a means of predicting the reaction model and comparing it with the theoretical master plots of *y*(*α*) and *z*(*α*), which can be obtained simply by transforming the experimental data [43]. As a result, the defined parameters are calculated and presented in Table 2 for pristine and AHP-loaded epoxy resin for all heating rates based on the previous DSC characterization. 

**Figure A4:**
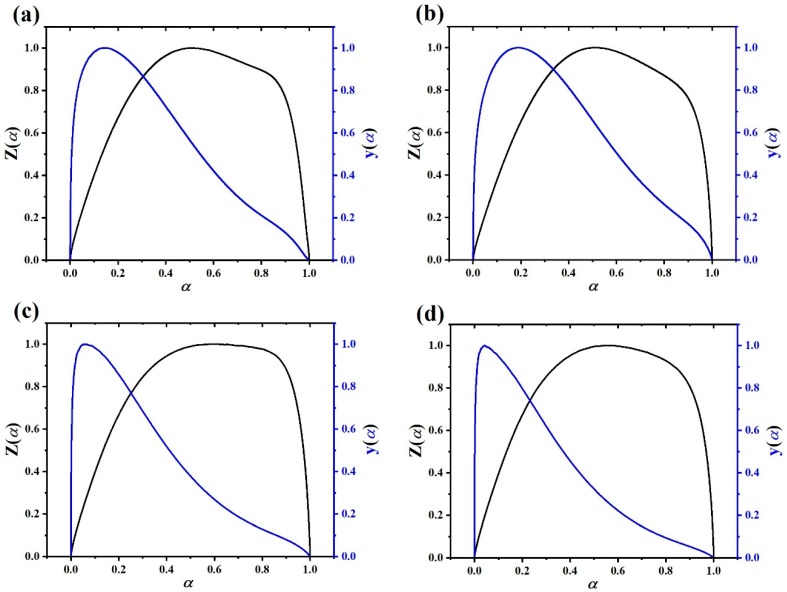
Variation of *y*(*α*) and *z*(*α*) versus conversion for prepared samples based on *Malek* model for (**a**) neat epoxy, (**b**) E-0.1AHP, (**c**) E-0.3AHP and (**d**) E-0.5AHP.

Interpreting the results of Table 2 shows that the values for *α_m_* are in all cases lower than those for *α_p_*. A maximum point exists in the *y*(*α*) profile (Figure A4 in Appendix B.2), as can be observed from the shape of *y*(*α*). Accordingly, two-parameter autocatalytic kinetic models fit the given condition [43].

The *Friedman* and *Kissinger* methods can also be used to determine the autocatalytic reaction model based on experimental data in the form of the equation below:

(5) $$\frac{d\alpha }{dt}=A\;exp(-\frac{E_{\alpha }}{RT})\alpha^{m}(1-\alpha ),$$

In Equation (5) *n* and *m* represents degrees of non-catalytic and autocatalytic reactions, respectively, and *A* is defined as a frequency factor, a measure of the molecular collision state. These parameters were calculated using the *Friedman* and *Kissinger* methods (Appendix C), and the results are summarized in Table 3.

The contents of Table 3 imply that the non-catalytic order of reaction (*n*) at all heating rates is first decreased due to the hindrance effect of nanoparticles in epoxy resin, while by increment of nanoparticle loading this parameter is increase to higher values compared to reference epoxy as a result of high reactivity of numerous AHP nanoparticles toward ring opening reactions, compromising the hindrance effect. This is also the case for the catalytic order of reaction (*m*) and frequency factor (*A*), for which a similar trend is observed. The increase in *A* values resulting from the introduction of 0.3 and 0.5 wt.% of AHP is further evidence of the physical impeding effect of nanoparticles, resulting in more energy being requiredfor the curing process. Moreover, the overall order of the cure reaction (*m + n*) is higher than 1 for most of the studied systems, revealing non-elementary aspects to be inherent to the cure reaction occurring in the nanocomposite systems [44]. The patterns described are predicted by both of the applied methods with a slight common difference, which is caused by the difference in approximation of the models.

**Figure 7:**
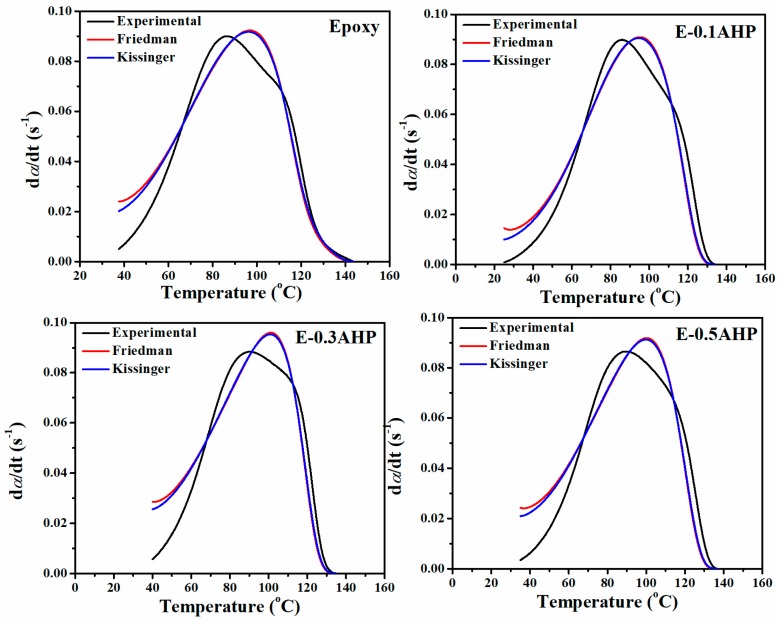
Comparison of experimental data with the kinetic models for the prepared samples at heating rate of 5 °C/min as a typical of computations done based on Friedman and Kissinger models.

The reliability of the models used in this study need to be assessed by a simple comparison of theoretical and experimental results of curing rate versus temperature which is depicted in Figure 7. The experimental curves show some discrepancies with respect to calculated values, revealing the complexity of cure kinetics of these systems. 

### 3.4. Glass Transition Analysis

Glass transition temperature for fully cured AHP/epoxy nanocomposites was measured in a heating cycle from 15 to 250 °C. The results were compared to the *T_g_* of cured bare epoxy and are provided in Table 4.

A brief review of the table provides an image of the molecular interactions of the constituents present in system. The glass transition temperature is slightly increased by the addition of 0.1 wt.% AHP nanoparticles to the polymer, which starts scaling down through the multiplicsation of the nanoparticle amount. It can be seen that the presence of low amounts of AHP reduces the simplicity of the molecular movements and hinders the segmental mobility of the polymer chains. This is because of the formation of a denser epoxy cross-linked network in favor of more active sites that react excellently with the epoxide group. However, when the concentration of AHP is increased to 0.3 and 0.5 wt.% of resin, although the number of active sites is significantly increased and, based on previous section, the curing progress of the system has been enhanced, the decrease of *T_g_* is a sign that indicates easier movement of molecules at imparted voids in proximity to the nanoparticles and resin. This could be due to the aggregation of nanoparticles and their relatively poor dispersion, which could undermine molecular mobility. In the case of 0.3 wt.% AHP/epoxy nanocomposite, the similarity of *T_g_* to that of neat epoxy shows the validity of this approach.

### 3.5. Thermal Stability

**Figure 8:**
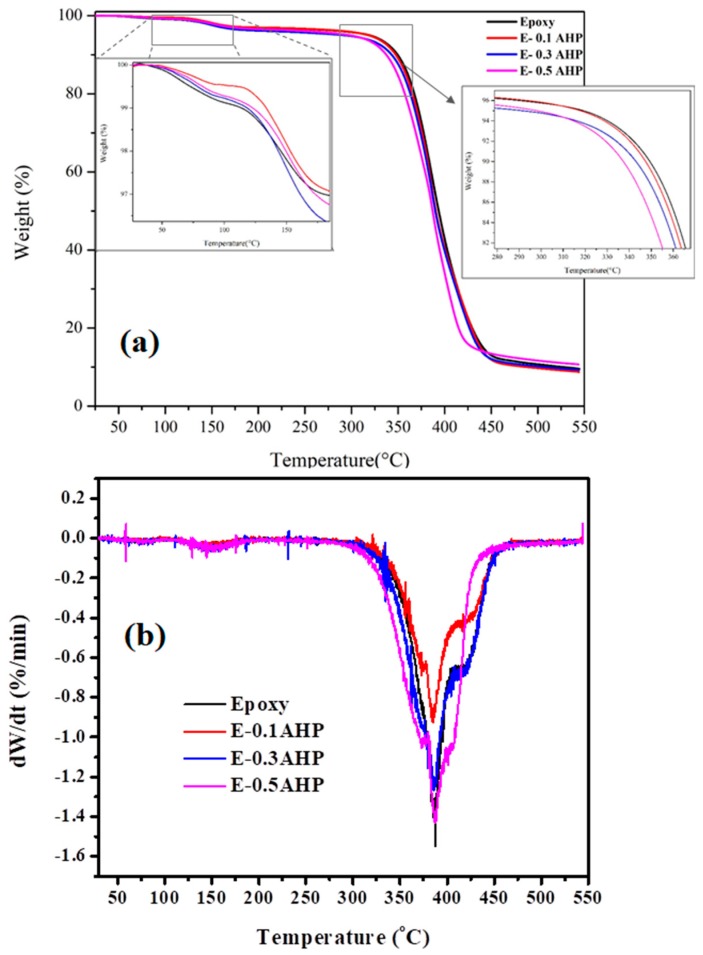
(**a**) TGA and (**b**) DTG thermograms of neat epoxy and its nanocomposites containing 0.1, 0.3 and 0.5 wt.% of AHP.

The thermal stability of AHP/epoxy nanocomposites in comparison to neat epoxy was determined by TGA under nitrogen atmosphere at a heating rate of 10 °C/min from room temperature to 550 °C. Figure 8 shows the dual-step TGA curves and Table 5 summarizes the characteristic data of thermal analysis. The early-stage degradation at around 100 °C can be related to solvent and small molecules which have lower thermal stability compared to epoxy structure [45]. Investigating the retarded thermal behavior of nanocomposites in this region, one can realize that addition of AHP nanoparticles to the resin decreased the decomposition rate, representing improved thermal resistance in the system, even at very low concentrations (0.1 wt.% of resin).

The decreasing trend of initial decomposition temperature (*T*_5%_) of nanocomposites (Table 5) demonstrates the higher activity of the O=P–O bond compared to the common C–C and C–O bonds in epoxy, which is boosted by increasing the nanoparticle content and leads to significant decline in *T*_5%_ (Δ*T* = 20 °C) to 300 °C for the highest loading [46]. In fact, the addition of AHP nanoparticles causes a decrease in the temperature at which the carbon layer forms [47]. However, this effect is reduced in the 0.1 wt.% AHP/epoxy nanocomposite due to the low amount of P-containing materials.

**Figure 9:**
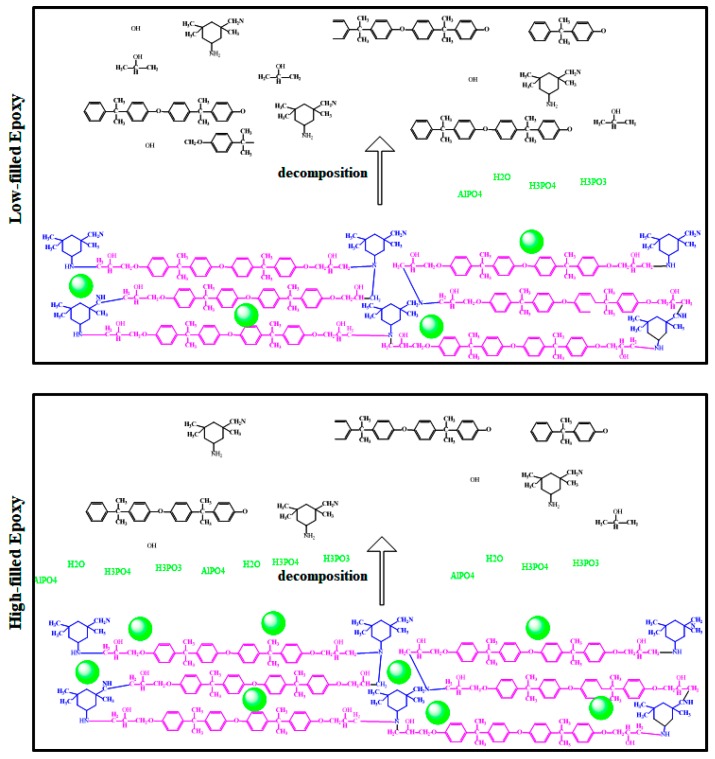
Possible decomposition mechanism of epoxy network in the presence of low (0.1 wt.%) and high (0.5 wt.%) AHP loadings.

The sharp drop in mass loss profile in Figure 8 at a temperature of around 350 °C can be ascribed to the decomposition of the epoxy resin, which is facilitated by the presence of phosphate compounds, catalyzing the decomposition process. Table 5 shows that the char yield values of the thermoset nanocomposites were more or less equal to that of the reference polymer, with a slight variation. It can be suggested that when the content of nanoparticles reaches a threshold (in this case, 0.5 wt.% of resin), AHP nanoparticles start to enhance char forming. Figure 9 proposes a schematic illustration of possible thermal stability induced by the incorporation of AHP into the epoxy matrix as a function of loading level. At low loading levels, it is anticipated that the presence of nanoparticles restricted the mobility of the epoxy chains. After the amount of AHP in the epoxy matrix reached the critical threshold of 0.5 wt.%, the release of volatile compounds and free radicals through epoxy network scission was made more difficult, hindering the diffusion of such low-molecular-weight molecules through the polymer matrix. Moreover, phosphoric acid, PH_3_ and AlPO_4_ released through the decomposition of aluminum hypophosphite [48]. At higher temperatures, phosphoric acid could be partly degraded to produce poly metaphosphoric acid, which could promote the carbonization of epoxy [49]. This effect is probably more likely at higher AHP loadings.

## 4. Conclusions

Aluminum hypophosphite (AHP) nanoparticles were produced through a milling process of AHP microparticles, and their epoxy nanocomposites were prepared in different loadings of 0.1, 0.3 and 0.5 wt.% with respect to the resin in order to investigate their potential for participating in the cure progress of thermoset epoxy resin. The shape and size of the fresh and milled particles was compared on the basis of SEM and TEM. The nonisothermal DSC of the prepared samples demonstrated that a denser cross-linked network of polymer was the case for the majority of nanocomposites due to the provision of a reactive Lewis acid element (Aluminum cation) in the system by the AHP nanoparticles. Comparing the experimental results of the nanocomposites with reference samples for qualitative cure analysis, the calculated *Cure Index* was found to be in the *Good* and *Excellent* states for all of the nanocomposites as a result of the extended length of the epoxy ring opening reaction. Additionally, the quantitative cure analysis was also performed based on a detailed protocol in order to investigate the kinetics of cure reaction in detail. The differential *Friedman* and integral *Kissinger* isoconversional methods were regarded to be predictors of kinetic parameters such as activation energy and order of autocatalytic and non-catalytic reactions. The results showed a decrease in the value of *E_a_* for low loadings from 72.5 (*Friedman*) and 62.4 (*Kissinger*) for neat epoxy to 64.6 and 54.9 kJ/mol., respectively. Agglomeration stemming from higher additive contents present in a system reduces the molecular mobility, and therefore increases the values of *E_a_*. Moreover, the *T_g_* characterization confirmed the higher crosslinking density, especially for low loadings of nanoparticles in resin, which results in an increase from 73.2 for neat epoxy to 79.5 for E-0.1AHP. The slight reduction of *T_g_* at high loadings was attributed to aggregation formation due to high surface energy. Finally, TGA analysis provided a context for the investigation of thermal stability of AHP/epoxy nanocomposites. Improved char forming ability was an achievement demonstrated by the analysis of E-0.5AHP. 

## Figures and Tables

**Table 1:** Cure characteristics of epoxy and its nanocomposites as a function of heating rate (*β*).

Designation	*β* (°C/min)	*T_onset_*	*T_p_* (°C)	*T*_endset_	Δ*T* (°C)	Δ*H*_∞_ (J/g)	ΔT*	ΔH*	CI	Quality
	5	26.8	86.9	143.7	116.8	179.9	n.a.	n.a.	n.a.	n.a.
Epoxy	10	27.7	98.8	139.9	112.1	192.5	n.a.	n.a.	n.a.	n.a.
	15	35.1	111.7	147.3	112.2	132.5	n.a.	n.a.	n.a.	n.a.
	20	40.6	118.42	150.5	110.0	145.9	n.a.	n.a.	n.a.	n.a.
E-0.1 AHP	5	19.0	86.5	134.5	115.4	232.8	0.99	1.29	1.28	*Excellent*
	10	26.9	102.3	143.9	117.0	201.2	1.04	1.05	1.02	*Good*
	15	36.4	115.6	149.6	113.2	181.7	1.01	1.37	1.38	*Good*
	20	41.2	123.4	156.2	115.0	165.2	1.05	1.13	1.19	*Good*
E-0.3 AHP	5	22.8	91.1	135.2	112.4	196.5	0.96	1.09	1.05	*Excellent*
	10	30.3	102.3	142.5	112.2	195.0	1.01	1.01	0.95	*Good*
	15	34.1	110.0	147.6	113.5	192.8	1.01	1.46	1.47	*Good*
	20	37.2	115.7	152.1	115.0	190.7	1.05	1.31	1.38	*Good*
E-0.5 AHP	5	22.3	90.6	137.0	114.8	250.5	0.98	1.39	1.36	*Excellent*
	10	28.1	101.0	145.8	117.7	213.5	1.05	1.10	1.09	*Good*
	15	34.3	109.3	149.0	114.7	199.7	1.02	1.51	1.54	*Good*
	20	35.5	113.3	152.8	117.3	172.2	1.07	1.18	1.26	*Good*

**Table 2:** The values of *α_p_, α_m_* and *α_p_^∞^* obtained from DSC analysis based on the *Malek* model at various heating rates.

Designation	Heating Rate (°C/min)	*α_p_* ^∞^	*α_m_*	*α_p_*
Epoxy	5	0.47	0.14	0.51
	10	0.70	0.15	0.55
	15	0.79	0.15	0.70
	20	0.83	0.17	0.79
E-0.1AHP	5	0.47	0.19	0.51
	10	0.59	0.18	0.59
	15	0.70	0.17	0.72
	20	0.72	0.18	0.72
E-0.3AHP	5	0.50	0.06	0.59
	10	0.57	0.07	0.60
	15	0.62	0.08	0.62
	20	0.65	0.08	0.64
E-0.5AHP	5	0.50	0.04	0.55
	10	0.54	0.05	0.58
	15	0.60	0.06	0.59
	20	0.62	0.06	0.57

**Table 3:** The curing kinetic parameters of the samples based on *Friedman* and *Kissinger* models.

Designation	*β* (°C/min)	*Ē*_a_ (kJ/mol)	ln *A* (s^−1^)	Mean (s^−1^)	*m*	Mean	*n*	Mean
Friedman								
Epoxy	5	72.5	22.2	22.0	0.40	0.39	1.17	0.94
	10		22.1		0.39		1.00	
	15		21.9		0.41		0.83	
	20		21.9		0.37		0.78	
E-0.1AHP	5	64.4	19.6	19.3	0.27	0.29	1.12	0.89
	10		19.3		0.31		0.91	
	15		19.2		0.31		0.77	
	20		19.2		0.26		0.74	
E-0.3AHP	5	77.2	23.4	23.3	0.53	0.49	1.03	0.93
	10		23.4		0.51		0.94	
	15		23.3		0.47		0.90	
	20		23.3		0.44		0.85	
E-0.5AHP	5	80.7	24.6	24.5	0.56	0.52	1.19	1.06
	10		24.6		0.54		1.09	
	15		24.5		0.53		0.98	
	20		24.5		0.46		0.97	
Kissinger								
Epoxy	5	62.4	18.9	18.8	0.26	0.25	1.08	0.87
	10		18.9		0.25		0.92	
	15		18.7		0.27		0.77	
	20		18.8		0.24		0.71	
E-0.1AHP	5	54.9	16.5	16.4	0.14	0.16	1.04	0.82
	10		16.4		0.18		0.83	
	15		16.3		0.18		0.71	
	20		16.4		0.13		0.69	
E-0.3AHP	5	69.3	21.0	21.0	0.43	0.39	0.97	0.88
	10		21.0		0.41		0.89	
	15		21.1		0.371		0.85	
	20		21.0		0.34		0.80	
E-0.5AHP	5	72.5	21.9	21.9	0.44	0.41	1.11	0.99
	10		21.9		0.42		1.03	
	15		21.9		0.41		0.92	
	20		22.0		0.34		0.91	

**Table 4:** Glass transition temperature of fully cured AHP/epoxy nanocomposites at *β* of 10 °C.min^−1^.

Sample	*T_g_* (°C)
Epoxy	73.2
E-0.1AHP	79.5
E-0.3AHP	73.1
E-0.5AHP	69.5

**Table 5:** TGA data of neat epoxy and AHP/EP nanocomposites.

Samples	*T*_5%_(°C)	*T*_10%_(°C)	Residue Weight Ratio at 550 °C
Epoxy	319	351	9.8%
E-0.1AHP	320	348	9.0%
E-0.3AHP	295	343	9.4%
E-0.5AHP	299	337	10.7%

**Figure A1:**
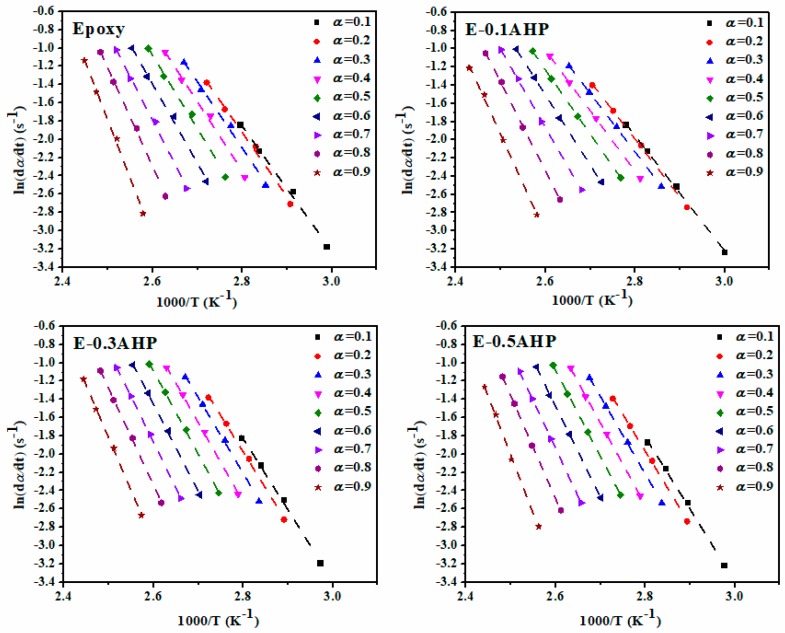
Plots of *ln*(*dα/dt*) vs. *1/T* of the prepared samples based on *Friedman* model at *β* = 5 °C/min.

**Figure A2:**
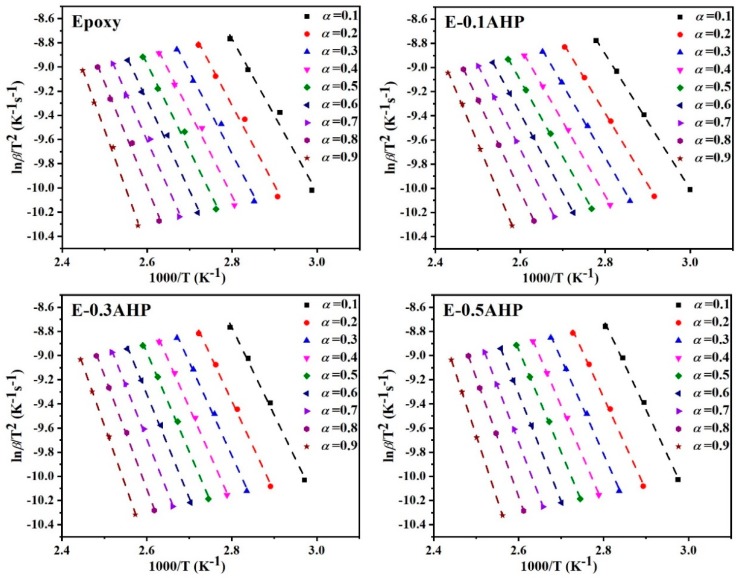
Plots of *ln*(*β*/*T*^2^) vs. 1/*T* for prepared samples based on the *Kissinger* model.

**Figure A3:**
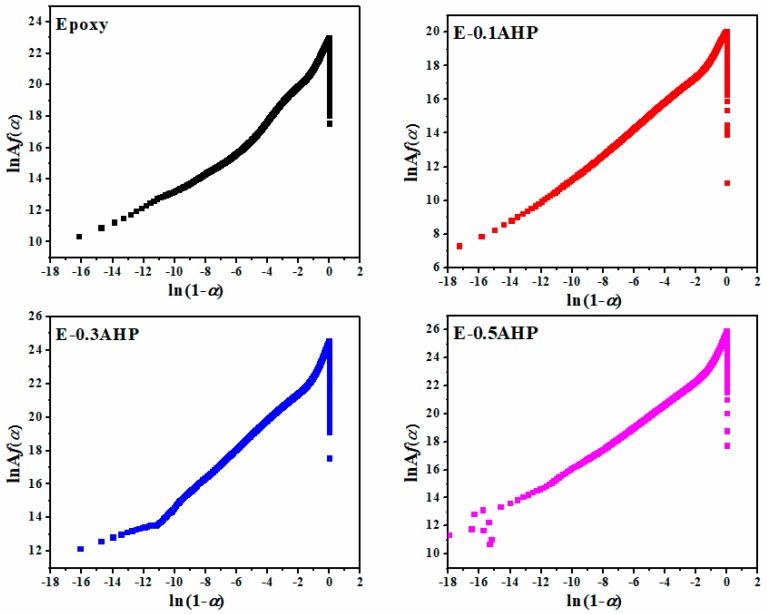
Plots of *ln*[*Af*(*α*)] vs. *ln*(1 − *α*)for the samples at heating rate of 5 °C/min used in calculation of activation energy via Friedman method.

**Figure A5:**
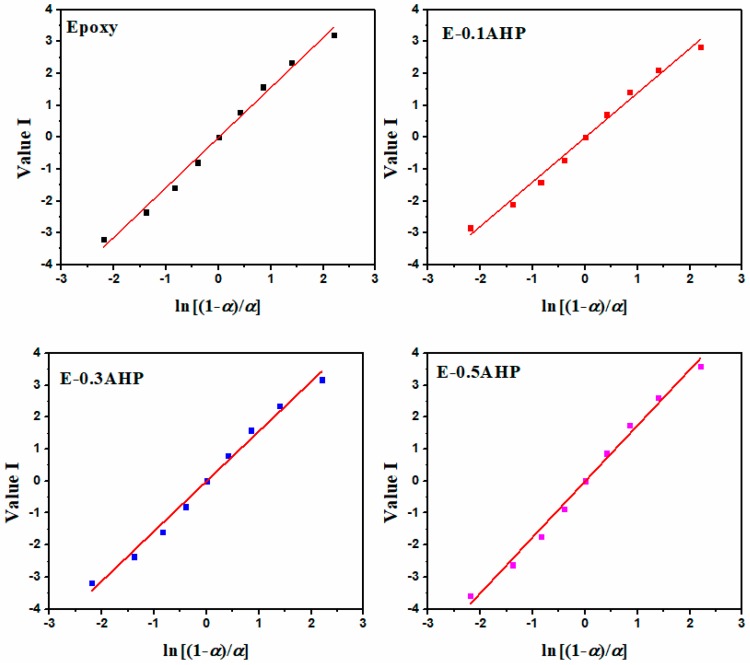
Plots of *Value I* calculated using DSC data for prepared epoxy system under heating rate of 5 °C/min.

**Figure A6:**
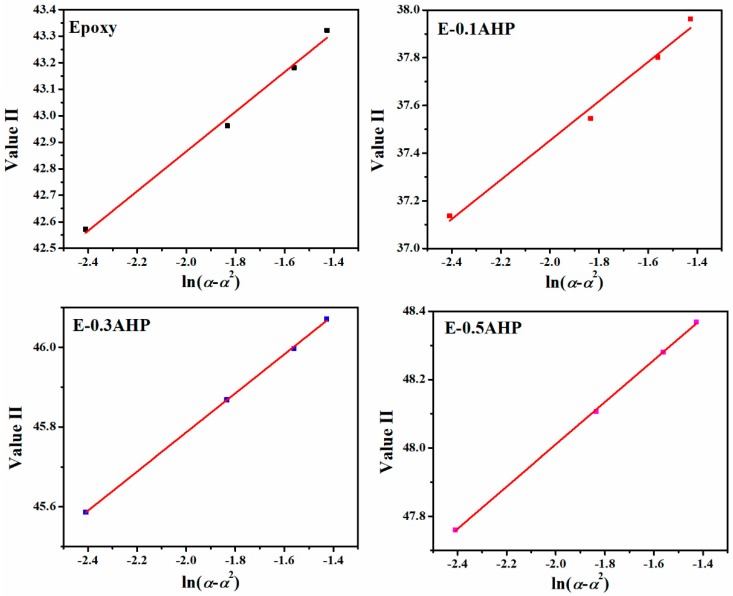
Plots of *Value II* calculated for prepared samples under heating rate of 5 °C/min.

## Appendix A. Isoconversional Kinetic Methods

### Appendix A.1. Friedman Model

According to the literature, *Friedman* model can be expressed as the following equation:

(A1) $$ln\left[\beta_{i}{\left(\frac{d\alpha }{dT}\right)}_{\alpha ,i}\right]=ln\left[f(\alpha )A_{\alpha }\right]-\frac{E_{\alpha }}{RT_{\alpha ,i}},$$

where *α*, *β* and *E_α_* are representative of cure conversion, heating rate and activation energy, respectively. Depicting the $\ln\left[\beta_{i}{\left(d\alpha /dT\right)}_{\alpha ,i}\right]$ vs. 1000/*T_α_*, one can find the values of activation energy at each conversion from the slope of Figure A1.

### Appendix A.2. Kissinger Method

*Kissinger* method can be defined by the following equation:

(A2) $$ln\left(\frac{\beta_{i}}{T_{\alpha ,i}^{2}}\right)=Const-1.0008\left(\frac{E_{\alpha }}{RT_{\alpha }}\right),$$

Plotting $ln\left(\beta_{i}/T_{\alpha ,i}^{2}\right)$ vs. 1000/*T_α_* gives the possibility of obtaining activation energy from the slope of straight lines showed in Figure A2.

## Appendix B. Curing Reaction Model Determination

### Appendix B.1. Friedman Model

Based on the *Friedman* method, the model of the epoxy curing reaction can be determined using Equation (A3). The shape of plot of *ln*[*Af*(*α*)] vs. *ln*(1 − *α*) denotes the deviation from nth-order reaction (Figure A3).

(A3) $$ln\left[Af(\alpha )\right]=ln\left(\frac{d\alpha }{dt}\right)+\frac{E}{RT}=lnA+nln\left(1-\alpha \right),$$

For the nth-order cure kinetics, a straight line should be obtained by plotting *ln*[*Af*(*α*)] vs. *ln*(1 − *α*), the slope of which gives the reaction rate (*n*).

According to the *Friedman* method, based on the shape of plot of *ln*[*Af*(*α*)] against *ln*(1 − *α*), the curing mechanism can be determined (Equation (A3)). From the shape of Figure A3, showing a maximum point out of the range of −0.51 to −0.22, it can be concluded that a disagreement between experimental and theoretical results exists, and the reaction mechanism of neat epoxy and its nanocomposites does not follow an autocatalytic model.

### Appendix B.2. Malek Method

The important parameters of *Malek* method *y*(*α*) and *z*(*α*) can be determined by Equations (A4) and (A5) as follows:

(A4) $$y(\alpha )={\left(\frac{d\alpha }{dt}\right)}_{\alpha }exp\left(\frac{E_{0}}{RT_{\alpha }}\right)=Af(\alpha ),$$

(A5) $$z(\alpha )={\left(\frac{d\alpha }{dt}\right)}_{\alpha }T_{\alpha }^{2}\left[\frac{\pi (x)}{\beta T_{\alpha }}\right],$$

The term in the brackets of Equation (A5) is negligible and ineffective on the shape of the *z*(*α*) function and can be omitted. In Equation (A4), the value of *E*_0_ is determined by isoconversional methods, that is, *Friedman* and *Kissinger* models in which the activation energy is determined from the slope of *ln*(*β_i_*) vs. 1000/*T_p_* plot as shown in Figure A1 and Figure A2. The experimental values of *y*(*α*) and *z*(*α*) for AHP/epoxy nanocomposite as a function of α are shown in Figure A4 and compared with theoretical master plots.

The values of *y*(*α*) and *z*(*α*) should be normalized as follows to take a value between 0 and 1:

(A6) $$y_{n}(\alpha )=\frac{y(\alpha )}{\max\;\left[y(\alpha )\right]},$$

(A7) $$z_{n}(\alpha )=\frac{z(\alpha )}{\max\;\left[z(\alpha )\right]},$$

The maximum values of, *y*(*α*)_max_ = *α_m_* and *z*(*α*)_max_ = *α_p_*, can be found from the following expressions, which are critical for kinetic characterization:

(A8) $$f^{\prime }(\alpha_{m})=0,$$

(A9) $$f^{\prime }(\alpha_{p})\;g(\alpha_{p})=-1,$$

The parameter *g*(*α_p_*) is the integral form of the reaction model.

## Appendix C. Determination of Degree of Reaction

The degrees of autocatalytic reaction (*n* and *m*) and the frequency factor (*A*), as essential kinetic parameters, can be determined through the following equations:

(A10) $$ValueI=ln\left(\frac{d\alpha }{dt}\right)+\frac{E_{\alpha }}{RT}-ln\left[\frac{d(1-\alpha )}{dt}\right]-\frac{E_{\alpha }}{RT\prime }=(n-m)ln\left(\frac{1-\alpha }{\alpha }\right),$$

(A11) $$ValueII=ln\left(\frac{d\alpha }{dt}\right)+\frac{E_{\alpha }}{RT}+ln\left[\frac{d(1-\alpha )}{dt}\right]+\frac{E_{\alpha }}{RT\prime }=(n+m)ln(\alpha -\alpha^{2})+2lnA,$$

The slope of the plots of *Value I* vs. *ln*[(1 − *α*)/*α*] (Figure A5) and *Value II* vs. *ln*(*α*− *α*^2^) (Figure A6) gives the value of (*n*− *m*) and (*n* + *m*), respectively, and from the intercept of the latter, the plot *A* value can be calculated.
